# Tuberculosis in pregnancy

**DOI:** 10.25122/jml-2021-0001

**Published:** 2021

**Authors:** Lucian Gheorghe Pop, Nicolae Bacalbasa, Ioan Dumitru Suciu, Paris Ionescu, Oana Daniela Toader

**Affiliations:** 1. Department of Obstetrics and Gynecology, Alessandrescu-Rusescu National Institute for Mother and Child Health, Bucharest, Romania; 2. Department of Obstetrics and Gynecology, Carol Davila University of Medicine and Pharmacy, Bucharest, Romania; 3. Center of Excellence in Translational Medicine, Fundeni Clinical Institute, Bucharest, Romania; 4. Department of General Surgery, Clinical Emergency Hospital of Bucharest, Bucharest, Romania; 5. Department of Obstetrics and Gynecology, Ovidius University, Constanta, Romania

**Keywords:** tuberculosis, infection, congenital

## Abstract

Tuberculosis (TB) in pregnancy is not only a matter of the past; it is also a current problem. These days, TB appears through mass migration and tourism in countries where it was believed that this condition is eradicated. Adequate knowledge about the medical history of patients, risk factors, diagnosis and treatment of tuberculosis should be part of the armamentarium of each physician involved in clinical practice. TB is mainly found in urban and socially deprived areas. Due to the length of the treatment, there is an increased risk of drug resistance in partially treated patients. Strong knowledge about the history, risk factors, diagnosis and treatment of TB should be part of the armamentarium of each physician. Many practitioners are reluctant to request a chest X-ray in pregnancy due to the fear of harming the fetus. Bypassing a diagnosis can have a devastating effect on the mother and fetus, as well as their family and medical staff. This article discusses the matters of diagnosis and treatment of asymptomatic infection and active TB in pregnancy.

## Introduction

According to the World Health Organisation (WHO), tuberculosis (TB) is one of the most common causes of death worldwide, with approximately 1.5 million people dying every year. In 2018, there were 10 million people infected with TB, of which 3.2 million were women, many of them of reproductive age [1]. The incidence of TB on pregnancy reflects TB incidence overall, and there is a huge gap between developed and underdeveloped countries despite a 2% decrease per year [1]. A wide variation exists even among high-income countries, which is related to ethnic minorities, such as differences between white, non-Hispanic (0.2/100000) and. Native Hawaiian/Pacific Islander (11.8/100000) population in the United States of America (USA) regarding women of fertile age [2]. Recent mass migration caused a TB relapse even in countries that rarely experienced TB cases, such as Sweden [3]. In the European Union, TB remains one of the significant health problems, with over 55537 cases reported in 2017. Romania has one of the highest incidences of TB, with 13004 cases reported [4]. Strong attention should be given to HIV-infected women, where TB incidence is ten times higher compared to HIV-negative women [5].

### Natural history

Looking back at the natural history of tuberculosis, the disease was discovered by Robert Koch in 1892, and its name means fungus (Gk. mykes) and bakterion (Gk. little rod) and derives from the Greek language [6].

**Figure 1.**
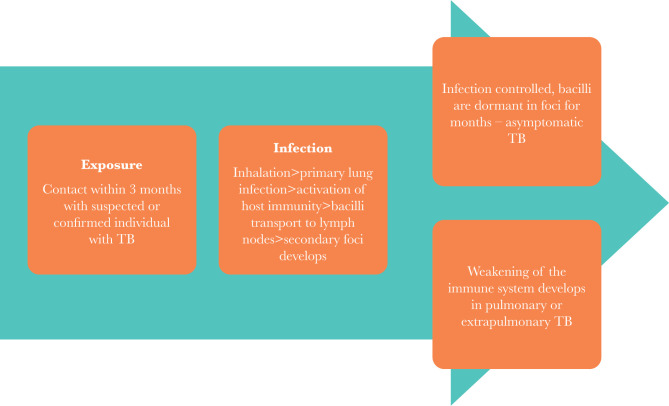
History of tuberculosis infection.

Mycobacterium tuberculosis is the most common bacteria encompassing 99% of the positive culture cases, while M. *boviis* and M. *Africanum* are accountable for only 1%. There are several other types, such as Mycobacterium *kansaii*, Mycobacterium *xenopi*, Mycobacterium avium, and Mycobacterium *fortuitum*, that rarely cause disease in humans but do give positive results on the Mantoux tuberculin skin test and can cause resistance in HIV and TB patients [7]. Figure 1 shows the natural history of TB in an infected individual.

### Risk factors for TB in pregnancy

The healthcare practitioner should keep in mind risk factors for TB, as screening for this condition could expedite the diagnosis. Screening in pregnancy is recommended by the World Health Organization (WHO) if the prevalence is higher than 100/100000 cases. This can be achieved in two ways: X-ray with protective shield and screening for symptoms compatible with TB [6], as shown in Table 1.

**Table 1.** Screening in pregnancy.

**Alvarez-Leon [8]**	Family member with infectious cases
**Starke, JR [6]**	Travel to countries where TB is common, i.e., developing Asian and African countries
**Janssens et al. [9]**	Living in ethnic minority communities where TB is common
**Gupta A [10]**	Having an immune system damaged by HIV or other health problems
**Mahendru A [11]**	Being extremely young or elderly, as the immune system is less robust at these ages
**Lewlyn [12]**	Chronic poor health and nutrition because of the standard of living; Living in deprived or packed houses

Clinical diagnosis can only be made as long as there is an active disease and features are similar to the non-pregnant state and cough (71%), weight loss (41%), malaise, fatigue (30%), fever (30%) are the most common [6]. The lungs are the most common TB site, followed by the lymph nodes, bone, kidney, gastrointestinal system, and very rarely genital area, including the breast [7]. The typical location of TB can be modified in HIV-positive women, with unusual locations such as and the meninges, mediastinum, and kidneys; also, tuberculosis is more serious in these sites [11].

### The effect of pregnancy on tuberculosis

It does not seem that pregnancy might have a harmful effect on tuberculosis as long as this condition is treated properly. The TB prognosis is influenced by the extension of the condition, the radiographic model, and women's susceptibility to the disease. Pregnancy can indirectly influence patients with tuberculosis since many of the TB symptoms overlap with typical pregnancy signs such as fatigue, tachypnea, which can delay treatment and diagnosis. However, approximately one-half and two-thirds of the patients are in the latent stage, making diagnosis even more difficult [7].

### The effect of tuberculosis on pregnancy

From reproduction to delivery and beyond, TB can influence all stages of the birth process. Infertility in the form of uterine synechia, tubal obstruction, implantation, and ovarian failure can all be consequences of TB. Even when pregnancy occurs, the risk of a tubal or abdominal pregnancy is higher than in unaffected women [13]. Prematurity and low birth weight risk increases by 2–3 times and perinatal death in babies from a mother with tuberculosis increases by 6 times [12, 14].

So far, there are no data regarding the true incidence of TB infection in pregnancy; estimations show around 500,000 children aged 0–4 are infected with TB. Congenital transmission can occur via the amniotic fluid, by hematogenous spread, or both. Once the placenta is infected, the fetus can acquire the infection through the umbilical cord following a primary site in the liver with subsequent hematological dissemination or via amniotic fluid when the primary complex is within the lungs or gastrointestinal tract [15, 16]. New-born acquired infection must be distinguished from congenital infection. Aerosolized transmission after birth will result in pulmonary disease and is not considered congenital TB. Cantwell *et al.* developed easy-to-apply diagnostic criteria, which are well-suited with modern practice (Table 2) [17].

**Table 2.** Congenital tuberculosis – diagnostic criteria.

Tuberculosis infection of the placenta or maternal genital tract
Hepatic primary complex or hepatic caseating granulomas
Lesions in the first week of life
Exclusion of postnatal transmission through investigation of contacts and strong guidelines compliance

### Congenital tuberculosis

Congenital tuberculosis occurs more frequently during pregnancy or delivery from primary maternal infection. In a study conducted by Peng *et al.*, 121 mothers out of 162 had no previous history of tuberculosis infection, which is consistent with the available literature data. Maternal diagnosis of tuberculosis is difficult, with the majority of women being diagnosed postpartum and after their children were diagnosed with congenital tuberculosis. Tuberculosis can affect mothers' wellbeing, as it was showed by a Norwegian study, in terms of preeclampsia, postpartum hemorrhage, and difficult labor [18]. Extrapulmonary TB does not influence the pregnancy but can cause recurrent admission and higher maternal mortality rates when the central nervous system is affected [19].

In newborns, tuberculosis symptoms such as fever, respiratory distress, hepatosplenomegaly, cough, poor feeding, failure to thrive, cyanopathy, abdominal distension are non-specific and overlapping with bacterial sepsis, or congenital viral infections, which makes the diagnosis even more challenging. Clinical signs do not improve after antibiotics and their condition can deteriorate. Laboratory findings are common in multiple diseases, and for that reason, we underline Cantwell's criteria regarding caseating hepatic granulomas and TB infection in the placenta [20].

A fast and robust diagnosis can be achieved these days using imaging techniques such as chest X-ray, ultrasound, computed tomography (CT), and magnetic resonance imaging (MRI) with imaging-guided sampling, allowing early treatment.

### Tuberculosis diagnosis in pregnancy

The test of choice in TB diagnosis is the tuberculin skin test (Mantoux test). Lately, the interferon-gamma release assay (T-spot) test that measures interferon-gamma is used as well. Pregnant women have their immune function suppressed, but this does not appear to influence the test result compared with the immunosuppressed patient due to various conditions such as (HIV, drug addiction) where 40–60% of the test results are false-negative. When there is a clinical suspicion of TB, a thorough pursuit should follow, involving a chest X-ray with shield even below 12 weeks of gestation if the patient's condition is highly suggestive of TB (Table 3) [20–22].

**Table 3.** Laboratory diagnosis.

**Investigation**	**Interpretation**
**Mantoux test – 0.1ml tuberculin skin test**	0–4 mm – no reaction; 5–10 mm – doubtfully positive; 10–15 mm – reactive in high risk cases; >15 mm – positive in all cases
**Chest X ray**	Nodular shadow in the upper zone; Loss of volume, fibrosis, cavitation; Primary focus in latent TB
**CT/MRI of the spine, chest, brain**	Depends on the site, useful for extrapulmonary TB
**Smear/culture sensitivity (sputum, tracheal aspirate, bronchial wash, cerebrospinal fluid)**	Gram-positive, acid-fast bacilli in culture
**Interferon-Gamma Release Assay – T-spot TB**	T- spot >6 – positive result

### Treatment and Prognosis

#### Preventive therapy

Isoniazid, which has no side effect on the fetus, is an effective treatment to prevent latent infection and active disease in susceptible individuals. The main shortcoming of isoniazid is its hepatic toxicity. The incidence of death due to isoniazid is 1/20,000. The American Thoracic Society recommends that for most women, preventive treatment should be delayed after delivery with the notable exception of an HIV-positive individual or contact with a contagious person. Preventive treatment should be delayed after the first trimester [21–24].

#### Management of drug sensitive-TB in pregnancy

Currently, TB therapeutic schemes are limited in providing evidence-based advice as pregnant women are usually excluded from trials due to fears of teratogenicity.

Pregnant women with pulmonary drug-sensitive-TB may be treated with either the standard four-drug scheme or a three-drug scheme without pyrazinamide and prolonging the treatment for 9 months. According to WHO, there is no specific treatment for TB in pregnancy but using pyrazinamide in pregnancy is under question in some countries due to the lack of safety data during gestation [25]. There are particular conditions when the benefits of using pyrazinamide outweigh the risks, such as in HIV/TB patients or those with extrapulmonary disease. Also, streptomycin should not be used in pregnancy as it causes ototoxicity. Vitamin K should be prescribed to the fetus at birth to reduce the risk of hemolytic anemia due to rifampicin.

#### Management of drug resistant-TB in pregnancy

The most challenging issue regarding TB treatment is drug-resistant tuberculosis. Second-line drugs may have a harmful effect on the fetus. Ethionamide, cycloserine are category C drugs that have showed teratogenic effects in animals. Aminoglycosides such as the aforementioned streptomycin or kanamycin can cause ototoxicity [26]. The fluoroquinolones are harming the growing cartilage and should be avoided entirely in pregnancy if possible. In drug-resistant TB, the treatment is usually prolonged to 24 months [24].

A complex issue is the management of HIV-positive patients during pregnancy, particularly for that category of patients not yet receiving antiretroviral therapy (ART). Multidisciplinary team management, according to the recent guidelines, is mandatory.

## Conclusion

Usually, tuberculosis is seen in endemic areas; nevertheless, the consequences of a missed diagnosis could be tragic, increasing the risk of miscarriage, perinatal death, or maternal death. TB should be suspected in any woman coming from an endemic area with non-specific symptoms and clinical signs. Also, pregnancy makes this condition even more challenging both in terms of diagnosis and treatment with limited guidance and data regarding second-line drugs in pregnancy. The present barrier in screening and diagnosis represents a risk for both mothers and healthcare professionals. Whether preventive therapy should be prescribed during the pregnancy or suspended until delivery still requires an answer. For this reason, future research should focus on drug safety, prevention therapy, and optimizing screening programs.

## Acknowledgements

### Conflict of interest

The authors declare that there is no conflict of interest.
